# We Specialists

**Published:** 1892-03

**Authors:** Richardson Grey

**Affiliations:** East Orange, N. J.


### "WE SPECIALISTS."[1]

BY RICHARDSON GREY, M.D., EAST ORANGE, N. J.

Mr. President and Gentlemen,—In accepting an invitation to read a paper before your society I did not anticipate the difficulty I should meet in attempting to fulfil my promise. In writing a paper to be read before a special audience there are two points to be considered if any degree of success is to be attained. The subject must be one with which the writer is well acquainted, and it must also be one in which his hearers have some interest.

You can readily appreciate the difficulty which has confronted me in observing these two essential points in complying with an invitation to address a dental society. Twenty years spent in

---

[1] Read before the Central Dental Association of Northern New Jersey, April 20, 1891.

medical study and practice make a subject in general medicine most agreeable to my own inclinations; but such a topic would have a very limited interest for you. It has been my fortune to be intimately associated with dental men in their practical work in the office for several years, but as an observer only.

The border-line between the work of the specialist in throat-diseases and the dentist is more sharply defined than would be supposed at first thought. For instance, in looking into a patient's throat the teeth are in full view, and yet I must confess that I do not often notice them. An artificial denture even escapes my attention unless it falls down and interferes with my work. And how often does the dentist see the tonsils which probably come into his view every day? The antrum of Highmore has sufficient interest to both of us to justify an article, and it was my original intention to use it for a subject. New developments in this direction have put a new phase of its discussion before us, but all that can be said about translumination has been so recently reported that it cannot be repeated to advantage at this time.

Taking these things into consideration, my mind was turned from the border-line of medical and dental practice into another channel. I am myself a specialist in medicine, and in the medical profession specialties are more important as the years go by. Moreover, echoes of certain articles read in dental journals were floating through my memory. The impression left on my mind by these wordy arguments as to whether dentistry is a specialty in medicine or a profession *de novo* is not clear as to the *casus belli.* Therefore let me say just here that it is not my intention to say anything on this subject, and, exercising whatever privilege the writer of an article may have to limit its scope, a taboo is rigidly placed on that subject at this time.

The time has gone by when the usefulness of specialties can be questioned. In all the arts, sciences, and professions the microscope is an important adjunct. Nothing but good can come from the minute and close study of any subject worth studying at all. The benefit is not only directly to the afflicted who seek relief, but also to those who practise, for with the advance of the specialty idea no one can hide ignorance under the cover of general knowledge and abstract statements. It is not the usefulness of specialties that is brought under discussion now, but their limitations. To what end are specialties to come? How fine are the subdivisions to be? Are we to have specialties within specialties, on the *imperium in imperio* style?

The eyes offer a wide field for study and special skill, but the ears have always been compelled to take a secondary relation to them. How long will it be before some man will give the ear its proper place as a separate specialty ?

The air-passages present a range of diseases sufficient to fully occupy the whole time of any man in study and practice, and yet are limited to an extent that permits the average mind to master the subject thoroughly and retain the knowledge from year to year. Are we ever to have men announcing themselves as specialists in the tonsils ? The fingers and toes offer a fairly good field for æsthetic and humanitarian practice, and it has, indeed, been filled in a certain way. But my curiosity has been often excited as to the outcome of manicure and chiropodist practice. It seems to be tending towards an evolution into something higher than it is now, and when this advance comes, will we see men graduating in medicine and announcing themselves as specialists in this branch of practice, or taking a brand-new degree of Doctor in Manicure and Chiropodist Work ?

Now, while these are merely imaginary results as yet in medicine, the dental profession presents us with an actual instance of what we have been considering. There is at least one item in dental practice which has claimed the dignity of a specialty. There may be more, but this one will serve for illustration. Attached to dental cards in the public print this legend may be sometimes seen : "Gas a specialty." I have often wondered what the exact meaning of that phrase is, and it is still an unfulfilled intention on my part to make inquiry about it at some favorable opportunity. Imagination, however, has been busy meantime, and many solutions have presented themselves for recognition.

Does Dr. Blank, who makes gas a specialty, mean that he has a special way of administering gas which is an advance on the usual methods ? Does he mean that the gas used is specially prepared for him, or is his own special preparation ? Does he mean that he has made a special study of gas and its use, so that he is better qualified for its administration than dentists usually are ? Or does he mean that he is one of the few dentists who use gas at all ?

If the mercy shown to those who make voluntary confession will be extended to me, and hoping that it will be, let me confess to you that the suspicion has sometimes forced itself upon me that, after all, this announcement of "gas a specialty" is something of the character of the agent used.

And yet—forbear with me—I also am a gas specialist! The years spent in "assisting" in the administration of nitrous oxide certainly entitle me to claim the distinction of special attainment, and, without a taint of self-exaltation, I can say that, as well as any man living, I know how to attach the necessary apparatus to the cylinder so that it shall not leak; I know how to turn the valve so that sufficient gas shall be liberated for the use of the patient, and the bag not burst; I know how to support the weight of the apparatus so that it shall not be unduly hard for the operator to keep the mouth-piece firmly over the face; long experience has made me perfect in avoiding the vent through which malodorous exhalations escape from the patient's lungs with each expiration; and last, but not least, I have especially learned to close the valve of the cylinder when the operation is completed, so that the small margin of profit from the gas given to the patient may not be lost by leakage from the cylinder. This is comprehensive, but it does not include the mental process going on, as it were, in pursuit of the gas as it enters the patient's lungs; the changes it causes and those through which it itself passes; the resultant exhalations, chemical formulæ; in short, all that vast store of knowledge which does not appear to the onlooker, present nevertheless, but not important to the patient nor vital to the operation. Now, having made so full and honest a statement for myself, may I ask my friend what he means when he announces "gas a specialty"?

When one has reached such a lofty plane of knowledge and experience in a specialty as has been just described, he begins to learn something of what it means to be charitable to those not so well informed. In reading dental journals I am sometimes grieved to find articles in which the writer holds up to ridicule general practitioners in medicine, because they have been found to be densely ignorant in dentistry when weighed in the balance of the severe critic. Now, leaving out the inquiry which might be justifiable as to what reason can be given why a medical practitioner should know more about dentistry than about ordinary carpenter-work, for instance, I have never read these statements without wishing that the lack of charity displayed might be corrected by a high grade of special knowledge. How often have such thoughts chased each other through the by-ways and avenues of my cerebrum while I have been assisting a gentleman duly authorized to put after his name the cabalistic letters signifying that by the grace of the State of New Jersey he is at liberty to pull teeth as a dentist. With the first snore from the victim, or, that failing, as it sometimes will, by sundry punches in the eye, or by raising an arm and letting it fall at the imminent risk of breaking a bone as it descends upon the arm of the chair, the moment that full "gas-phyxiation" has arrived is decided. The inhaler is dropped and the operator plunges into his work of extraction. Suddenly, in a most unmistakable manner, the patient makes known the return of consciousness; while the operator, his work unfinished, exclaims petulantly, "Pshaw! how fast he comes out of the gas!" My soul, sitting serenly in the exalted attitude attained by special knowledge, murmurs in echo, "How fast!" We specialists are nothing if we are not charitable.

But there is a serious side to this specialty question which deserves all the time that can be given to its discussion. The first point for consideration is as to what, properly speaking, may be said to constitute a specialty. There is a use made of the word which, whatever argument may be advanced in its favor from a philological stand-point, cannot be recognized as expressing the idea of a specialty, as it is now generally understood by professional men. In my preceding remarks I have attempted to ridicule this erroneous idea. This is a use of the word which has, in the past, been the basis upon which the prejudice against a specialist has been founded. In that sense specialist and quack are synonymous, and it is a matter for regret that this prejudice has not entirely disappeared from professional ranks.

My own conclusion as to what may properly be called a specialty is this: Any organ, or group of organs, may be said to be a proper field for a specialty, in the broad sense of the word, when it furnishes in itself, in pathological conditions, sufficient material to fully occupy the time of a man who devotes himself to a study of its diseases and the application of remedies to their relief. It should be possible also for a man of ordinary ability to master all the details of principles and their application to that particular subject, and by mastery of details is not meant that mental congestion so often observed in students when they appear for examination. It has been commonly remarked that a medical student, and possibly it is true of the dental matriculate also, knows more when he graduates than he ever does after. Mastery of details means that mental assimilation which makes the knowledge part of the man, so that he can at any time take up any feature of the subject without needing to refer to authorities. It is positively refreshing to meet such a man and hear him speak on his specialty.

It is evident that medicine, as taught in the seven branches necessary for graduation with the degree of M.D., is too vast for any but a phenomenal mind to master and retain in details. This is now fully recognized in the post-graduate schools where the special branches of medicine are taught, and attention to minute points is given to supplement the general teaching of the college.

My observation of dentistry leads me to think that it does not labor under the disadvantage of comprising too vast an amount of material for mastery by an individual, and therefore it is, in the broad sense, a specialty. This statement is not intended to convey an invidious meaning, and has no reference to dentistry in its relation to medicine or anything else. Am I wrong in saying that the average man can—not easily, as some would seem to suppose, but by diligent application—master dentistry in its details, and yet not have so vast a store of knowledge that he cannot carry it into practice without being unduly burdened, or becoming too top-heavy for satisfactory progress in the work? If this be so, there cannot be, in a legitimate sense, a specialty within dentistry. The dentist who is not master of his profession in all its phases is without excuse. He cannot plead ignorance, for there is no excuse for ignorance. He should not consider himself qualified for his profession because he can put in a gold filling as well as any one else. It is positively disheartening to hear men talk as though they were everything that any dentist can be, because they can pull a tooth or put in a good filling. If that is all there is to dentistry, would it not be a mere trade?

There are important principles underlying every detail of dental work; there are pathological processes going on in the tissues with which the dentist deals; there are more things than the macroscopic vision can see; and these things are of essential, even vital, importance to the best work. The man who is not qualified in these respects will quarrel with this, and claim that his fillings are just as good as can be done, and that teeth never go back on him. But, then, mistakes never happen to the ignorant. Physicians in general practice do undoubtedly often put drugs into the stomach without knowing why they do so; but if dentistry is in this broad sense a specialty, a dentist should never put medicine into or around a tooth without knowing why he does so.

Being myself a specialist, I hold out a hand of welcome to a dentist as being a fellow-specialist. My purpose in these remarks is not to read any one a lecture, but to stimulate us to greater proficiency. Five years of special work, following fourteen of general practice, have demonstrated to me its value, and I am desirous that we shall maintain so high a standard that the demonstration shall reach all classes.

There still remains for consideration the broad question of the status of the specialist. In medicine it is now demanded that a man practising a specialty shall first receive the degree of M.D. In fact, the medical profession have always demanded this; but in these latter days the State is interfering in this matter, and aiding the profession to give the man who claims to be a specialist in the proper sense protection from the quackery which has gathered about the idea in the past. There was a time when the profession expected that a man would give some practical attention to general medicine before entering upon his specialty, but that seems to be changing now.

The point of most importance in this connection is, that while the profession keeps so close a hold of the man up to a certain point, it then withdraws at the most critical time. There is an utter absence of control over the man who enters upon the practice of a specialty as to whether he is properly qualified for that branch. Take the eye, for example,—a most important and, at the same time, delicate organ. If one makes a mistake in treating it, and then is compelled to remove it, the artificial eye will not in any sense take the place of the natural, and it would be impossible to persuade the patient that it was superior to the one removed; that it would not be troubled by dust getting into it, for example. A man receives his degree of M.D., and decides to make the eye his specialty. The stock of knowledge of this organ which he brings with his degree is very limited and must be increased. He studies, but it is the business of no one to find out how well he studies. The broad foundation has been carefully laid under proper direction, but the superstructure has had no superintendence. He may be well or ill prepared, who knows? I was about to say, who cares?

Theoretically, the degree of doctor in medicine covers the practice of every organ in the human body; but when it comes to special work in one organ, or any set of organs, there is found one instance, and one only, in which any degree of supervision is enforced. In connection with the teeth alone is there an assurance demanded that qualification exists. I have myself tested this matter and know it to be so. The registry of a medical diploma does not suffice, unless the special declaration of dentistry is also made. Now an advanced step has been taken, and examination takes the place of declaration. This is as it should be, and I for one am not complaining. I would like to see the same hedge raised around every organ of the body that is made a special subject for practice.

On the other hand, a man cannot treat specially any part of the body without first laying a broad foundation of knowledge of every other part, with one exception again. He can be licensed to treat the teeth although his previous education has been so limited that it has not passed the rudimentary stage. Please do not understand me as saying that all dentists are uneducated. My meaning simply is, that while the hedge has been raised about dentistry (and this I believe to be right), very serious gaps have been left which should be filled, as doubtless they will be. In justice to the mass of dentists, this also should be said: that the ignorant, uneducated man who secures a license to practise dentistry cannot be a specialist such as has been described, nor properly represent the dental profession.

We are drifting along in this matter of professional education just as the currents of circumstances may chance to carry us, and it does seem as though the twentieth century should witness the inauguration of some plan by which there should be systematic, thorough, and universal teaching, so that from the time a boy takes up his letters to the time when he takes up his life-work there should be systematic intent, and at every important stage he should be called upon to prove his competency up to that point. To some extent it is done, but at vital points there is omission. This is one of the benefits "we specialists" receive from the microscopic study of a subject, that we appreciate more and more the blessedness of thoroughness.

